# Migrants’ Quarantine and COVID-19 Pandemic in Italy: a Medico-anthropological View

**DOI:** 10.1007/s42399-021-00993-2

**Published:** 2021-06-17

**Authors:** Silvia Di Meo, Enrico Bentivegna

**Affiliations:** 1 DISFOR–Department of Education Sciences, University of Genoa, Genoa, Italy; 2 Internal Medicine and Emergency Medicine, Sant’ Andrea Hospital, Sapienza University, Rome, Italy

**Keywords:** COVID-19, Quarantine, Migrants, Medical anthropology, Public health

## Abstract

The COVID-19 pandemic represents an important risk factor for migrants’ health. Paul Farmer highlighted the risk of global health response in emergency conditions exacerbating global and social inequalities. We argue that this is the case for quarantine ships and migrants’ management during the pandemic. Every aspect of infection-control and prevention measures acquires detention characteristics in these situations. With emphasis to the evolution of the doctor-patient relationship and to the anthropological and cultural aspects that were established during the pandemic, this article aims to provide an integrated view where physicians and anthropologists collaborate to deepen the understanding of the topic.

## Introduction

The ongoing COVID-19 pandemic represents a major risk factor for migrants’ health, both for the increased risk of getting infected and for the negative health outcomes related to barriers in accessing health services [1–3]. Migrants’ travel conditions are associated with several risk factors for mental and physical well-being [4, 5]. Fear of deportation, xenophoby, social stigmatization and cultural differences caused by discriminatory policies lead to migrants delaying access to health services [6] or entirely distancing themselves from them [7]. Furthermore, in the event of an emergency, socio-cultural differences are exacerbated, and a combination of economic, political and social causes limits access to welfare service and legal rights [8]. Indeed, legal associations have criticized the biopolitical management in detention camps and in quarantine ships. Given that migrants’ management in a pandemic context must have ramifications across medicine, anthropology and jurisprudence, this paper explores the topic from a medico-anthropological point of view.

## Migrants’ Healthcare During Pandemic

**Fig. 1:**
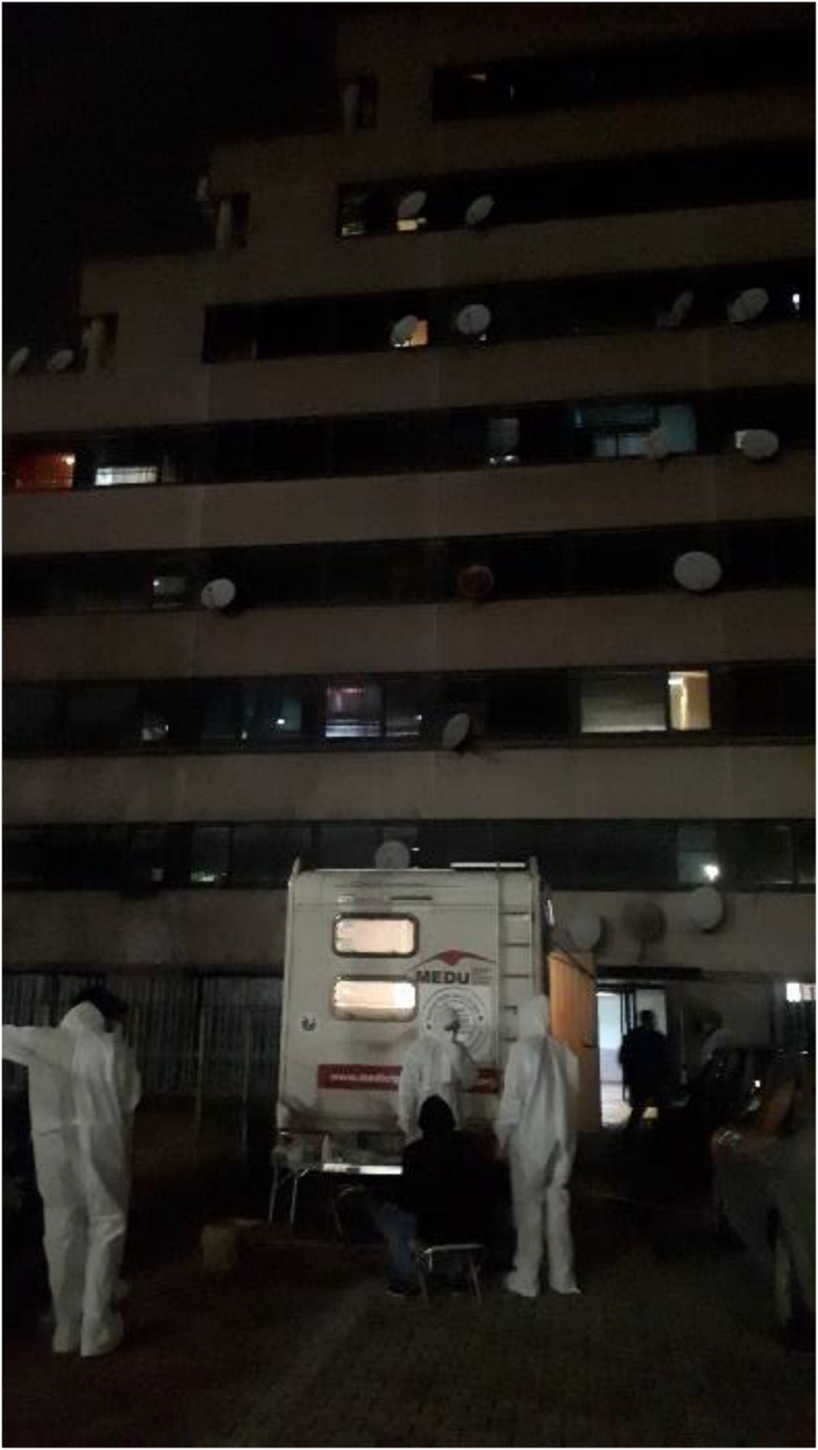
MEDU staff during his assistance for COVID-19 prevention and monitoring in informal settlements, Rome, Italy

Despite global pandemic efforts to protect population and improve health, resources have limited the deaths of native people in rich countries; the same cannot be said for migrants. Preventive measures often fail in camps and detention centres where millions of migrants and refugees live in appalling conditions worldwide. Furthermore, it was reported that migrants dying with Coronavirus Disease 19 (COVID-19) are younger than natives [9]. Across the Mediterranean area, ten thousand people live in crowed camps without adequate infrastructure. In these conditions, physical distancing and preventive measures are unfeasible. Among migrant workers living in overcrowded accommodation, the second pandemic wave took hold [10]. In a Lancet comment, World Health Organization (WHO) leaders advocated for increased awareness of refugees’ and migrants’ conditions in humanitarian settings. Conditions in work or in custodial sites have been reported as inadequate to guarantee health right and security for COVID-19 prevention. Non-governmental organizations (NGOs) that work in these contexts have reported such critical issues [11]. Without any efficient COVID-19 plan enforced by governments, NGOs often try to compensate for health care system’s deficits. Several Italian associations highlight challenges that migrants face during the pandemic [12]; in a letter to the Minister of the Interior, a number of associations denounced critical conditions and solicited the closure of detention centres [13]. ASGI (Associazione per gli Studi Giuridici sull’Immigrazione—Association for Legal Studies on Immigration) sent a letter to ASLs (Aziende Sanitarie Locali—Local Sanitary Public Companies) to urge the need for a review of quarantine arrangements and management of migrants in CPRs (Centri di Permanenza per il Rimpatrio—Permanence Centres for Repatriation). Legal Clinic of Rome and other associations request the re-evaluation of migrants’ detention in terms of legal appropriateness [14]. In ref. [15], the Human Rights and Migration Law Clinic overviewed European detention centres with particular emphasis to health rights. Worthy of being mentioned is a recent work by the University of Oxford [16], investigating the problems associated with migrants’ detention centres during an emergency [16]. The criticism is often raised by organizations providing health assistance. In Greek islands, Médicins san Frontières has demanded evacuation of thousand asylum seekers to suitable accommodation. In Rome, MEDU (Medici per i Diritti Umani, Doctors for Human Rights), an independent, non-profit humanitarian organization, assists and monitors migrants during the pandemic, witnessing the condition of hygienic and social discomfort (Fig. 1).

## Quarantine Ships

The first major quarantine enforcement dates back to the Black Death pandemic of the fourteenth century. Giorgio Agamben defines it as a “state of exception” where all ordinary political, social, religious and economic activities are suspended. Health and political power subordinate human rights in the name of a state of emergency. Quarantine ships are reminiscent of such state of exception. As already happened and analysed in Liberia during the Ebola epidemic [17], daily activities became heavily scrutinized, and human rights were demoted in the name of the health emergency. Quarantine brings out several issues related to ethics and human rights [18–22], while its efficacy as infection-control measure remains controversial [23]. According to the definition reported by Centers for Disease Control and Prevention’s (CDC) [24], the term “quarantine” is referred to “separation and restriction of the movement of people who were exposed to a contagious disease to see if they become sick”. It is important to highlight how even the term “quarantine ship” itself is misleading since the rationale of these infrastructures is based on isolation of people who have not been in contact with any established case.

Paul Farmer highlighted the risk of global health response in emergency conditions exacerbating global and social inequalities [25]. This is the case for quarantine ships and migrant’s management during the pandemic. Every aspect of infection-control and prevention measures applied to the normal population acquires detention characteristics in migrants.

Quarantine ships, which operate as unofficial migrant detention centres, emerged in Italy starting from April 2020. Motivated by sanitary reasons, migrants were placed in these structures for monitoring the presence or the onset of symptoms compatible with COVID-19. It is possible to recognize these ships as characteristic of biopolitical management of migrants during the pandemic. There are several reports indicating how these ships are in fact operating as “floating hotspot” rather than sanitary presidia [26–28]. It was pointed out that many migrants do not know the reasons why they are in these floating centres. There is often no communication between healthcare personnel and patients, neither regarding the tests and swab results, nor regarding the diagnostic procedures. In these centres, lack of information generates a sense of frustration for patients, leading to an increase of calls for action by international human rights bodies and civil groups [29]. Worthy of interest and often the only source of data are reports from activist associations highlighting conditions of these informal settlements or detention centres.

“Really pitiful scenes: we saw these families waiting on the quay at 2:00 a.m., mothers with children, exhausted people – an operator remembers -. They didn’t explain anything to them, we provided them with information. Everyone was afraid of getting on the ship to be repatriated, which was absurd.” [30]. (Translation by authors).

Quarantine ships become an emblematic example where all the difficulties and the exceptionality of the emergency status are exacerbated.

Several elements suggest that migrant subjects are coerced into a passive attitude as a result of these processes. Data emerging from official and non-official sources underline the total lack of patients’ information during the quarantine. Lack of communication, linguistic and socio-cultural barriers in an exceptional and emergency context such as the quarantine ships produce a sense of frustration and withdrawal from doctor-patient relationship. Ethical issues aside, the biological benefits related to correct and detailed information regarding medical procedure are well recognized [31, 32].

“It is not an hospital ship. Health care over there is not what you can get on land. When the Open Arms group arrived on board, we swabbed people and separated them in the best possible way. But it was obviously not enough: it happened that people moved in shared space, contagion was always possible.” [30]. (Translation by authors).

“People could have been better isolated on land, and also better assistance could have been provided. But no, they had to be in the middle of the sea. It is a mediatic, theatrical isolation.” [30]. (Translation by authors).

The combination of European policies and emergency guidelines leads to an exacerbation of dissension between health personnel and migrants. In the name of the state of emergency, there has been a revision of the fundamental rights regarding health and patient’s management, which modern medicine upholds.

## Conclusions

In this paper, we attempted to highlight how during the pandemic the weakest categories are often more affected by difficulties related to the state of emergency. With a particular focus on quarantine ships, we explored the critical issues arising in migrant detention centres. The term “patient” derives from the Latin word “patior” and means to experience affections and emotions. This term is often surrounded by a devaluing and negative halo, referring to a subject with no bargaining power [33, 34]. During the last century, this concept has been progressively overcome by developing a medical-patient relationship based on dialogue and informed consent [35]. We stressed how the biopolitical management of migrants’ during emergency status generated a regression of this relationship. In these confined infrastructures, we recognized a demotion of the doctor-patient relationship from “shared decision-making” model to the more archaic, Hippocratic and paternalistic dimension that was overcome over the last century. Such paternalistic dimension has always been a one-sided relationship in which the doctor decides every aspect of therapy. The patient was not the subject of the treatment but the object of it [36]. Doctors were the absolute holder of knowledge. Decisions, preferences and opinions of the “incapable” patient, in a position of extreme inferiority, are not to be taken into consideration [36, 37]. Adopting a medico-anthropological vision, we analysed several official and non-official references trying to explore the critical issues arising in emergency health facilities—such as quarantine ships—during the emergency. There are numerous red flags that point to such a dangerous demotion of the above relationship. Emergency in these contexts becomes the justification of the law of biomedicine. The benefits of a shared doctor-patient relationship are well known, and it is no coincidence that more and more often there are protests by migrants within these contexts. With this article, we hope to stress the importance of not forgetting this aspect, even more so during a state of emergency. The virus disregards all borders [38]; COVID-19 prevention measures must not overlook migrants.

## Declarations

### Registration of Research Studies

NA.

### Guarantor

Enrico Bentivegna, MD.
